# A Clinical Approach to Multimodality Imaging in Pulmonary Hypertension

**DOI:** 10.3389/fcvm.2021.794706

**Published:** 2022-01-18

**Authors:** Christine Farrell, Aparna Balasubramanian, Allison G. Hays, Steven Hsu, Steven Rowe, Stefan L. Zimmerman, Paul M. Hassoun, Stephen C. Mathai, Monica Mukherjee

**Affiliations:** ^1^Division of Medicine, Johns Hopkins University, Baltimore, MD, United States; ^2^Division of Pulmonary and Critical Care Medicine, Johns Hopkins University, Baltimore, MD, United States; ^3^Division of Cardiology, Johns Hopkins University, Baltimore, MD, United States; ^4^Division of Radiology, Johns Hopkins University, Baltimore, MD, United States

**Keywords:** pulmonary hypertension, echocardiography, computed tomography, scintigraphy, magnetic resonance imaging

## Abstract

Pulmonary hypertension (PH) is a clinical condition characterized by progressive elevations in mean pulmonary artery pressures and right ventricular dysfunction, associated with significant morbidity and mortality. For resting PH to develop, ~50–70% of the pulmonary vasculature must be affected, suggesting that even mild hemodynamic abnormalities are representative of advanced pulmonary vascular disease. The definitive diagnosis of PH is based upon hemodynamics measured by right heart catheterization; however this is an invasive and resource intense study. Early identification of pulmonary vascular disease offers the opportunity to improve outcomes by instituting therapies that slow, reverse, or potentially prevent this devastating disease. Multimodality imaging, including non-invasive modalities such as echocardiography, computed tomography, ventilation perfusion scans, and cardiac magnetic resonance imaging, has emerged as an integral tool for screening, classifying, prognosticating, and monitoring response to therapy in PH. Additionally, novel imaging modalities such as echocardiographic strain imaging, 3D echocardiography, dual energy CT, FDG-PET, and 4D flow MRI are actively being investigated to assess the severity of right ventricular dysfunction in PH. In this review, we will describe the utility and clinical application of multimodality imaging techniques across PH subtypes as it pertains to screening and monitoring of PH.

## Key Points

- Pulmonary hypertension is a devastating disease and early detection improves morbidity and mortality.
- Echocardiography, computed tomography, nuclear imaging, and magnetic resonance imaging are non-invasive imaging studies for screening, classification, prognostication, and monitoring of pulmonary hypertension.
- New non-invasive imaging techniques such as strain imaging, 3D echocardiography, dual energy CT, and 4D flow MRI are emerging techniques that can assist in the diagnosis and monitoring of pulmonary hypertension.

## Introduction

Pulmonary hypertension (PH) is an insidious, highly morbid, and heterogeneous disease that is characterized by elevations in pulmonary arterial pressures and is classified into five groups based on etiology (1–3). Early diagnosis and referral are associated with better clinical outcomes, however the time from symptom onset to diagnosis is often greater than 2 years (4–6). PH is exclusively diagnosed using confirmatory invasive right heart catheterization (RHC) to measure mean pulmonary artery pressure (mPAP), pulmonary capillary wedge pressure (PCWP), and pulmonary vascular resistance (PVR) (7). Currently, PH is defined by a mPAP >20 mmHg, a threshold which was recently decreased from ≥25 mmHg based on epidemiologic data demonstrating the distribution of mPAP among healthy individuals and the significant impact of mildly elevated pulmonary pressures on morbidity and mortality (8).

While RHC is the only method to directly measure pulmonary and right heart pressures, it is invasive, resource intensive, and carries procedural risk (9). As a result, in 2015, the European Society of Cardiology/European Respiratory Society guidelines recommended the use of a variety of non-invasive imaging modalities to screen and risk stratify patients (10). The standard of care for screening and classifying PH includes transthoracic echocardiogram (TTE), chest computed tomography (CT), ventilation perfusion (VQ) scan, RHC, and increasingly cardiac magnetic resonance imaging (CMR). Multimodality imaging is useful for screening, classifying, prognosticating, and monitoring effectiveness of therapy in PH. This review seeks to describe the current imaging modalities used in diagnosing and monitoring the various forms of PH along with several novel imaging modalities that may soon be incorporated into clinical practice.

## Methodology

We conducted a search utilizing Medline/PubMed from November 1989 to June 2021 to identify relevant articles. Search terms included: pulmonary hypertension AND echocardiography OR magnetic resonance OR computed tomography OR nuclear OR cardiovascular imaging. Identified articles were then evaluated, including screening of references. Review articles, meta-analyses, and major medical society guideline documents were also assessed. Finally, selected articles were included if felt to be relevant in the authors' opinion. Data from these articles were abstracted and guided this narrative review.

## Results

We identified 46 articles on echocardiography, 19 on computerized tomography, 7 on nuclear medicine techniques including scintigraphy, and 45 on magnetic resonance imaging in PH.

### Echocardiography

**Figure 1:**
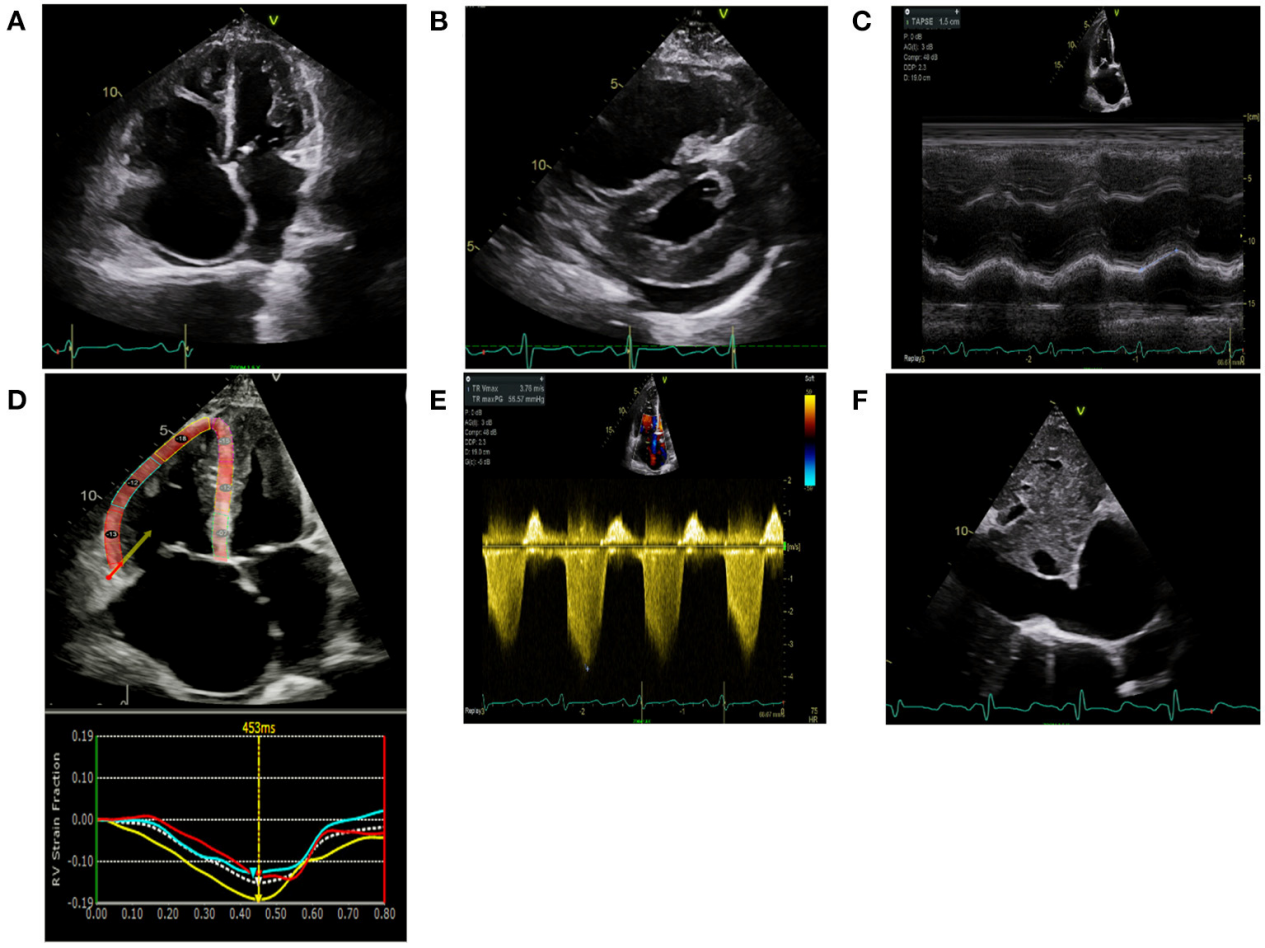
Echocardiographic images are shown in a scleroderma patient with severe pulmonary hypertension on stable therapies. **(A)** Apical 4 chamber view demonstrates severe right atrial enlargement with bowing of the interatrial septum from right to left suggestive of elevated right atrial pressures. The right ventricle is severely dilated and hypertrophied with a prominent moderator band. The left ventricle is hypertrophied and small. **(B)** Parasternal short-axis is shown in the same patient with marked RV enlargement and evidence of RV pressure overload distorting the normal circular short-axis geometry of the LV. There is a small posterior pericardial effusion present. **(C)** Tricuspid annular plane systolic excursion (TAPSE) utilizes M-mode techniques to measure the longitudinal motion of the basal right ventricular wall segment during systole as an estimate of right ventricular systolic function. TAPSE is mildly reduced at 1.5 cm (normal >1.6 cm) however fractional area change was 24% (moderate-severely reduced). **(D)** Right Ventricular Longitudinal Systolic Strain (RVLSS) is a recent echocardiographic advancement based on ultrasound-myocardial tissue interactions. Each segment of the RV in this example corresponds with a strain curve with the white dotted line representing an average of the segmental strain for the regional curves in this view. Regional RV free wall strain is reduced in the basal and midventricular wall segments with less reduction in the apical segment. Global strain is an average of the three RV free wall segments and is −14.3%. **(E)** Right Ventricular Systolic Pressure utilizes the peak tricuspid velocity to calculate the peak right ventricular systolic pressure using the modified Bernoulli equation. RVSP= [peak gradient (mmHg) = right atrial pressure + (4 × Peak velocity 2)]. In this example, RVSP = 57 mmHg + 15 mmHg = 72 mmHg. **(F)** Right atrial pressures are estimated from the IVC diameter made in subcostal view at end-expiration. In this example, the IVC is severely dilated at 3.2 cm with minimal respiratory variation suggestive of markedly elevated right atrial pressure of 15 mmHg.

TTE is the most common imaging modality used to screen for PH and is the mainstay for screening, monitoring of therapeutic response, and prognostication (11). As most deaths from PH are from right heart failure, recognizing the presence, and quantifying the degree of right heart dysfunction, is helpful in monitoring disease progression and prognostication. In addition to conventional two-dimensional (2D) TTE, speckle-tracking strain imaging and three-dimensional (3D) echocardiography are more specialized techniques that are increasingly becoming part of the standard of care in monitoring right heart structure and function. Representative echocardiographic images are shown in Figure 1.

#### Screening for Pulmonary Hypertension

Screening for PH using conventional TTE primarily relies upon assessment of the right ventricular systolic pressure (RVSP), which is measured from the tricuspid regurgitant (TR) jet velocity and size/collapsibility of the inferior vena cava (IVC) to estimate right atrial pressure (RAP) (12). Using the modified Bernoulli equation, RVSP = 4V^2^ + RAP with V equaling the maximum TR jet velocity (13). For RVSP measurements > 40 mmHg, a right heart catherization is recommended (14). However, RVSP has been shown in numerous studies across various PH subgroups to poorly correlate with systolic pulmonary artery pressure (sPAP) measured by RHC. These studies have routinely shown that RVSP is ± 10 mmHg different to the true sPAP in approximately 50% of cases (15–18). Additionally, the ability to capture and quantify the TR jet velocity can be technically challenging and is estimated to be feasible in only 75% of cases (19). RVSP can be combined with other non-invasive measures to evaluate the need for a RHC in suspected PH (20). Additional RV hemodynamics can also be obtained including PA end-diastolic pressure using end-diastolic pulmonic regurgitation peak velocity, mean PA pressure, and pulmonary vascular resistance. Lastly, early closure of the pulmonic valve due to rapid pressure equilibration of the RV and PA in midsystole can be detected using both M-mode or pulse waved Doppler signal, known as the “flying W” sign (21).

#### Assessment of the Right Heart

The American Society of Echocardiography has standardized measurements of right-sided cardiac structure and function (13). Measurements include the right atrial and ventricular area, fractional area change (FAC) as a surrogate of right ventricular ejection fraction (RVEF), tricuspid annular plane systolic excursion (TAPSE), RVSP, and the presence of a pericardial effusion. A right atrial area measured at the end of systole >18 cm^2^ has been independently associated with elevated right ventricular (RV) end-diastolic pressure (RVEDP) and mean RAP with a sensitivity of 89% and specificity of 82% (13, 22). The RV diameter at the base is considered enlarged when it is >42 mm. However, this measure only weakly correlates with the gold standard RV volume assessment *via* CMR (23, 24). Measurements based off estimations of the 2D RV area or volume, such as FAC or RVEF, are similarly flawed when compared to CMR techniques (25, 26) due to the complex shape of the right ventricle (27). Eccentricity index, or interventricular septal morphology, is a useful echocardiographic tool and assesses the interventricular dependency of the RV:LV from the parasternal short-axis view and is an important component of the ESC/ERS recommendations for PH screening (11). The presence of RV hypertrophy may also be seen in chronic pressure/volume overload states.

Due to the inaccuracy of RV area and volume assessments using 2D echocardiographic techniques, other measurements are used to estimate RV function. Tricuspid annular plane systolic excursion (TAPSE) measures the movement of the tricuspid annulus toward the apex between diastole and systole in M-mode. A measurement ≤ 1.7 cm is considered abnormal (28). TAPSE has been shown to closely correlate with RVEF on CMR and RHC (29). However, TAPSE measurements should be interpreted with caution in patients with severe TR as they have been shown to be less accurate in that setting (30). The Tei index, or myocardial performance index (MPI) of the RV, is measured using either color or tissue Doppler imaging and is a ratio of isovolumic time, both in contraction and relaxation, to ejection time (31, 32). Systolic wave velocity (S′) is another measure of myocardial contraction measured from tissue Doppler imaging and has been validated in an epidemiologic study of healthy individuals to define normal values (33). Abnormal tissue Doppler S′ velocity is defined as <9.5 cm/s.

#### Prognostication

As right heart failure is the primary cause of death among individuals with PH, assessment of abnormalities in the right ventricle by echocardiogram offers significant prognostic information. RA area and estimation of right atrial pressure have been demonstrated to be associated with mortality secondary to right heart failure (34). RVSP has been found to be an independent predictor of mortality in PH (35, 36) and while neither sensitive nor specific, the presence of a pericardial effusion has been shown to predict mortality in PH patients (34, 37, 38).

Recently, the REVEAL registry has included echocardiographic assessment of pericardial effusion in prognostic risk assessment of PAH (REVEAL risk score). Regarding RV functional assessments in individuals with known PH, reduced TAPSE has been shown to have a nearly four-fold increased risk of death (39) with every 1 mm decrease in TAPSE increasing the unadjusted risk of death by 17% (40). Myocardial performance index is associated with clinical status and mortality, as well as change in clinical status over time in response to therapy (31, 41).

#### Speckle-Tracking Echocardiography (Echo Strain Imaging)

Strain imaging is being increasingly incorporated into clinical practice as a measurement of RV systolic function (42). Strain (ε) is the deformation of cardiac tissue from an applied force with ε = (L_systole_-L_diastole_)/L_diastole_ with L being length (42) and multiplied by 100 resulting in a percentage of myocardial deformation across the cardiac cycle. A positive number indicates lengthening, and a negative number indicates shortening. Strain imaging provides a feasible non-invasive technique to assess cardiac mechanics for the detection of subclinical ventricular dysfunction.

Using 2D echocardiographic techniques, there are two methods by which strain can be calculated: tissue Doppler imaging (TDI) and speckle tracking echocardiography (STE). TDI-derived strain calculates the rate at which a particular segment of the myocardium moves toward or away from the transducer (43). TDI is less commonly used since it is highly angle dependent and requires high frame rates. In contrast, STE is angle-independent and performed by measuring the movement, or deformation, of ultrasound pixels over the cardiac cycle. It is particularly helpful in the right heart as it tends to preferentially measure speckles at the endocardial border whose longitudinal fibers account for 80% of RV contraction. STE-derived strain can be reported across the RV free wall regions or as an average of visualized segments known as global longitudinal strain (GLS) and is expressed as a percentage and a more negative number signifies a more shortening of the myocardial segment during systole. Worsening strain refers to a less negative number (a lower absolute value) than expected or diminished deformation along the longitudinal axis. GLS typically represents the basal, midventricular, and apical RV free segments however it may also include the basal, midventricular, and apical segments of the interventricular septum. The latter approach, however, is less favored due to inability to isolate RV and LV contributions (42). The most common measurement of strain in the RV is GLS, however individual longitudinal segmental strain is also being investigated in PH (44).

Reduced RV function using STE GLS imaging predicts worse clinical outcomes such as right heart failure and death in PH across various subgroups (45–47). Additionally, a reduction in RV free wall strain has also been shown to predict worse outcomes in PH (48). Reduced strain is one of the earliest signs of RV dysfunction as patients with less longitudinal deformation had worse outcomes than matched controls with equivalent right heart dimensions and TAPSE (49, 50).

For a strain analysis to be done, 2D echo image quality must also be adequate at a frame rate of at least 70–90 frames per second. Strain imaging requires post processing using dedicated software and can be performed utilizing CMR-based techniques as well. Echo-derived strain requires specialized software and ultrasound machines, which may result in increased cost, however can typically be performed during real-time image acquisition with minimal increase in patient exam time or retrospectively on previously acquired images. There is also a significant learning curve in strain analysis as automated endocardial border definition must be verified manually by experienced operators (51). Additionally, there is well-described vendor-specific variability in strain measures (52) and the cutoff values for normal and abnormal strain also depend on the analytic software and modality, i.e., CMR vs. echo-derived strain, being used. Longitudinal strain monitoring must therefore ensure that patients' images are analyzed using the same software across time and should be performed by experienced operators.

#### Three-Dimensional Echocardiography

3D echocardiography is a state-of-the-art imaging strategy increasingly being used in clinical practice (53). Estimations of the RVEF have been found to be more closely correlated to those measured by CMR (54–57). However, 3D echo tends to underestimate the true RVEF (58). Despite this, the accessibility of 3D echo is greater than CMR which makes this an attractive alternative. In addition, strain imaging has been combined with 3D echo to accurately predict RVEF (59). 3D imaging can be performed during both 2D and transesophageal echocardiography and is recommended in the assessment of severe TR (60) for grading and determining suitability for intervention.

### Chest Computed Tomography Imaging

**Figure 2:**
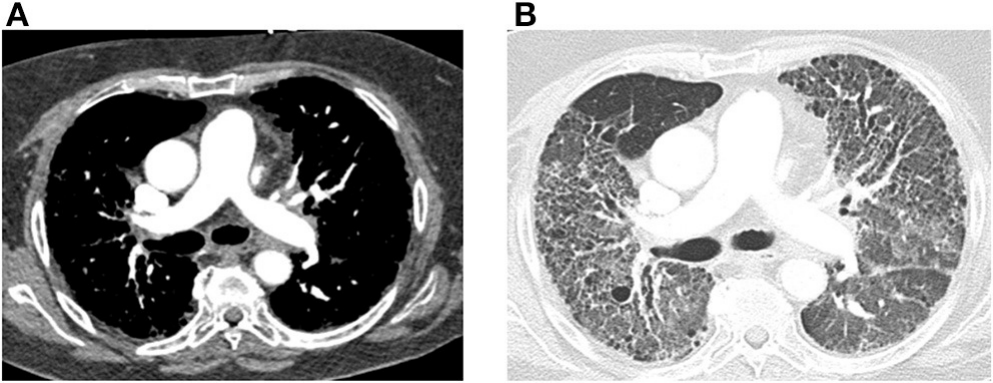
Computed tomography (CT) images of the chest with and without contrast are shown from a 64-year-old female with connective tissue disease, severe interstitial lung disease, and mixed severe pulmonary hypertension are shown. **(A)** Transaxial images are shown demonstrating an enlarged main pulmonary arterial size at 3.2 cm when compared to ascending aorta size of 2.9 cm at the same level suggestive of pulmonary hypertension. There is no evidence of pulmonary embolism with optimal contrast opacification. **(B)** Transaxial images in the lung window demonstrate extensive bilateral diffuse groundglass opacities and honeycombing. There is associated intralobular and interstitial thickening and bronchiectasis consistent with patient's known history of connective tissue disease associated non-specific interstitial pneumonitis.

Acquiring a non-contrast chest CT scan is part of the standard workup for the diagnosis of PH (10). The presence of lung disease on a chest CT along with abnormalities on pulmonary function tests can indicate PH secondary to lung disease (Group 3 PH). Along with its evaluation of the pulmonary parenchyma, there are several findings that can screen for PH on CT. These include the absolute size of the main pulmonary artery and its relative size compared to the aorta. Chest CT with contrast is also essential if acute pulmonary embolism is suspected as an etiology of PH. New CT techniques such as dual energy CT are also being investigated to measure lung perfusion qualitatively and quantitatively. A representative image from a patient with connective tissue disease- associated interstitial lung disease and mixed PH is shown in Figure 2.

#### Pulmonary Artery Size

The diameter of the main pulmonary artery (mPA) and its size in comparison to the ascending aorta correlate to mPAP on RHC. In the Framingham Heart Study, the 90th percentile for mPA diameter measured by CT was >29 mm in men and >27 mm in women (61). Subsequent work has shown that a mPA > 29 mm is correlated with elevated mPAP with a sensitivity and a specificity of ~80% and an *r* of 0.6 (62–66). A ratio of the mPA/ascending aorta >1 also correlated with elevated mPAP with ~70% specificity and sensitivity. The mPA size can be enlarged in fibrotic lung disease which can confound its use as a screen for PH in patients with these disorders. CT has not been shown to predict PH as accurately as echo or CMR (67, 68) but its sensitivity and specificity increase when it is combined with these modalities for screening (69).

#### Dual Energy CT

Dual energy CT (DECT) is a technique that acquires CT angiographic (CTA) images of the pulmonary vasculature at two different energy levels after the administration of intravenous iodine-based contrast. Due to the different attenuation properties of iodine contrast at these two different energy levels, the quantity of iodine inside the pulmonary vasculature, which can serve as a surrogate for pulmonary perfusion, can be isolated and measured. As CT scans are commonly used in the work up of PH, DECT has the capability to be built into the screening chest CT without extra radiation (70). DECT is primarily used as a replacement for the V/Q scan in diagnosis of CTEPH, but has also been investigated as a screening tool for PH and a tool to assess the degree of PH. DECT has been shown to have an 80% sensitivity in the diagnosis of CTEPH compared to VQ scintigraphy (71–74) which is much improved compared to standard CTAs (75). While this is the most useful and well-understood utility of DECT, additional assessment of pulmonary perfused blood volumes (PBV), representing the total amount of iodine inside the pulmonary vasculature at a certain timepoint, can be qualitatively and quantitatively used to screen for PH. Patients with PH have a mosaic attenuation pattern on DECT given the dysregulation of the pulmonary vasculature inherent to the disease (76). Additionally, the total degree of PBV has been shown to correlate with mPAP (77) along with the ratio of PBV to the attenuation of the pulmonary artery (78, 79). However, many of these findings are non-specific.

### Scintigraphy and Nuclear Imaging

#### Ventilation-Perfusion (V/Q) Scans

V/Q Scintigraphy is part of the standardized diagnostic workup of PH, specifically for diagnosis of WHO Group 4 chronic thromboembolic pulmonary hypertension (CTEPH) (10). CTEPH is defined as PH in the presence of mismatched perfusion defects by V/Q scan as well as signs of thromboembolism on CT and/or pulmonary angiography following 3 months of therapeutic anticoagulation (10). This modality is considered to be the standard of care in the initial evaluation for PH etiologies due to high sensitivity and specificity in the diagnosis of CTEPH, outperforming CTA alone (80–82).

#### Nuclear Medicine Techniques

**Figure 3:**
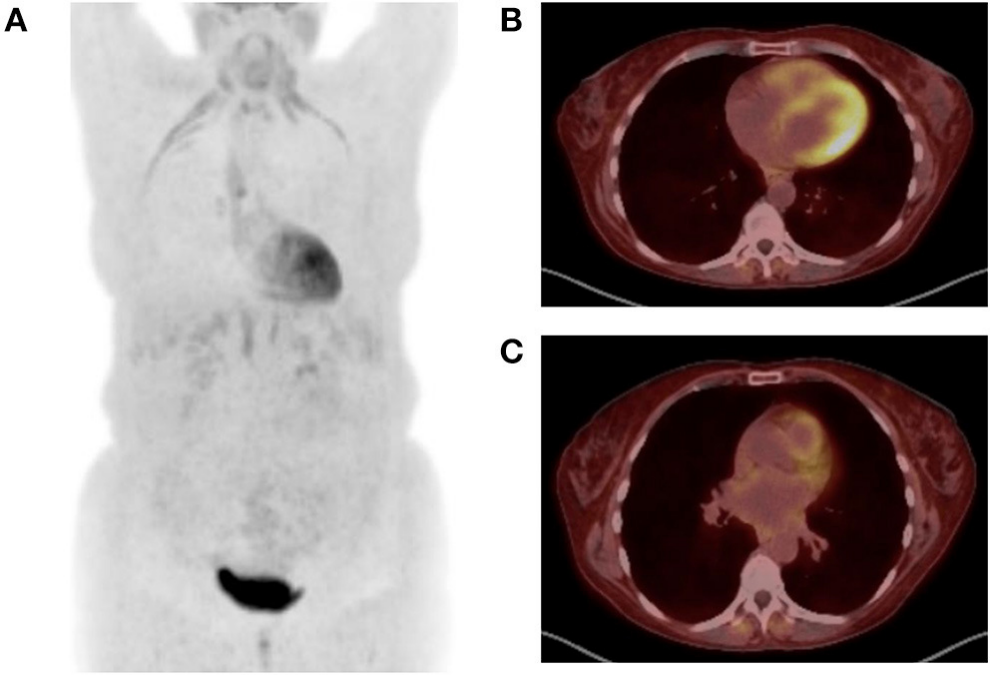
Positron emission tomography (PET) images are shown from a 52-year-old woman with emphysema and associated Group 3 pulmonary hypertension presenting with acute exacerbation. 9.78 mCi ^18^F-FDG injected at 119 mg/dl blood glucose level. Image acquisition 57 mins post injection. **(A)** Maximum intensity projection image demonstrates FDG uptake in the diaphragm, infrahyoid muscles, and intercostal muscles consistent with increased work of breathing noted during examination. There is also diffuse subcutaneous uptake, reflecting treatment with corticosteroids during the exacerbation. **(B)** Transaxial images at the midventricular level demonstrate abnormal uptake in the right ventricle. **(C)** Transaxial images at the level of the main pulmonary artery (mPA) demonstrate enlarged mPA and abnormal FDG uptake in the right ventricular outflow track.

Increased stress on the right heart in PH results in an increase in myocyte glycolysis and can be measured with a radioactively tagged glucose analog and measured by PET. Increased 2-deoxy-2-[18F]fluoro-D-glucose (FDG) uptake in the RV is observed in patients with PH and correlated with mPAP (83–85). Increased FDG uptake has been found to be associated with clinical worsening and death, and patients who respond to therapy show decreased FDG uptake over time (86, 87). In addition, alternatives to FDG, such as a radiotracer targeting mannose receptors on macrophages, have been similarly observed to detect PAH and respond to pulmonary vasodilator therapy (88). Further, hybrid PET/MRI imaging has demonstrated that a combination of RV ejection fraction and tracer uptake was associated with clinical deterioration or death in PAH patients (89). Figure 3 demonstrates representative FDG-PET imaging from a PH patient with emphysema.

**Figure 4:**
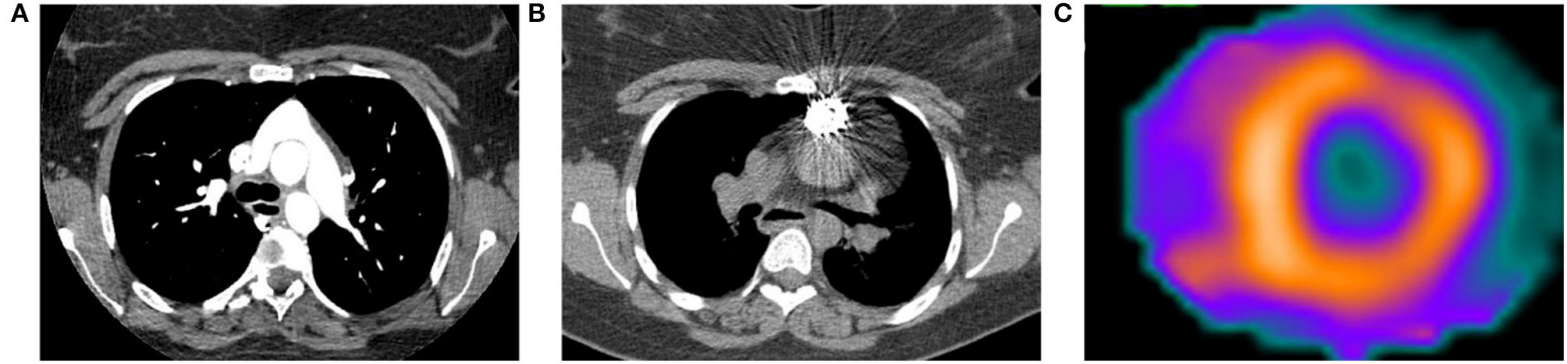
Computed tomography (CT) and ^99m^Tc-sestamibi single-photon emission computed tomography (SPECT) images from a 23-year-old woman with history of D-transposition of the great arteries (D-TGA) status-post repair. **(A)** Transaxial CT angiogram image demonstrating the characteristic appearance of the pulmonary artery and aorta after repair of D-TGA. **(B)** Non-contrast CT acquired at time of SPECT shows a stent in the pulmonary artery that was placed after the patient developed severe pulmonary artery stenosis. **(C)** Short axis SPECT image shows normal radiotracer distribution in the left ventricle with extension of uptake into the visualized portion of the right ventricle, consistent with pulmonary hypertension.

Single-photon emission computed tomography (SPECT) utilizes multiple different radiotracers to evaluate cardiac perfusion and function. Analogous to PET, patients with PH will have evidence of thickening, enlargement, and metabolic derangement in the RV. The most commonly used radiotracers in modern cardiac SPECT are mitochondrial imaging agents (e.g., ^99m^Tc-sestamibi), and their increased uptake in the RV is reflective of both increased RV mass and increased energy production and use (90). Figure 4 is from a patient with a pulmonary artery stenosis and increased ^99m^Tc-sestamibi uptake in the RV.

### Cardiac Magnetic Resonance Imaging

#### CMR Quantitative Assessment of Structure and Function

**Figure 5:**
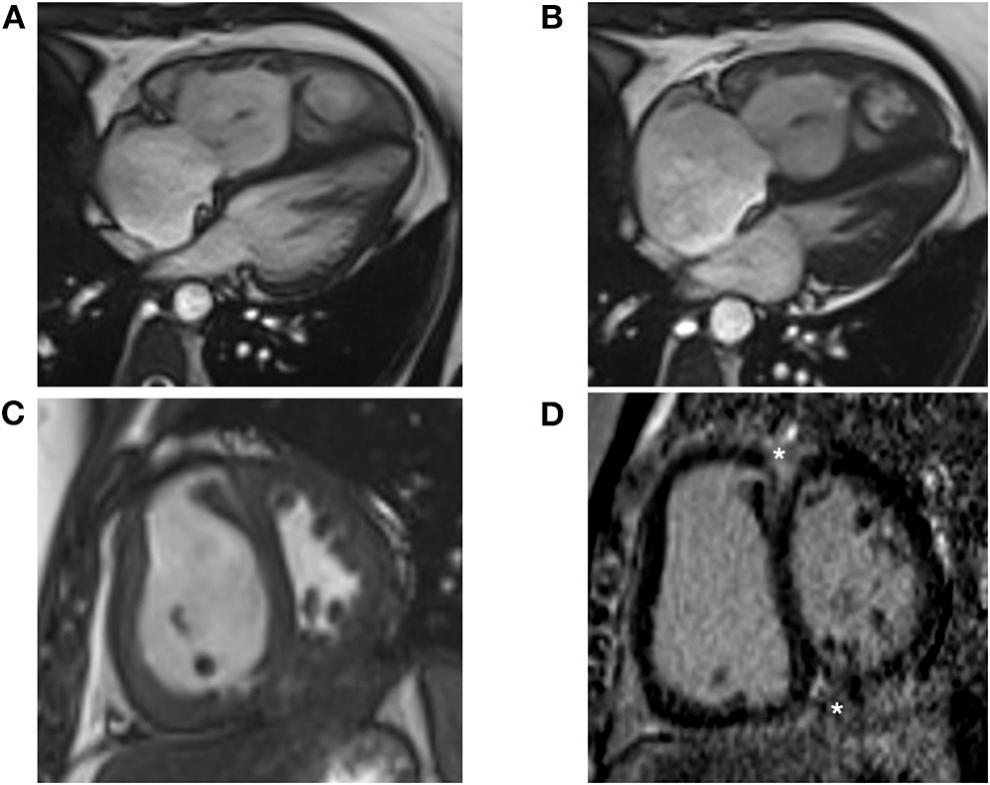
Cardiac Magnetic Resonance (CMR) images are shown from a 38-year-old female with idiopathic pulmonary arterial hypertension. **(A)** Four-chamber bright blood CMR image from end diastole shows a dilated and hypertrophied right ventricle at a mean pulmonary pressure of 47 mmHg. End systolic images show leftward bowing of the interventricular septum from elevated right ventricular pressure. **(B)** Late systolic images show leftward bowing of the interventricular septum from elevated RV pressure. **(C)** Short axis CMR image shows marked hypertrophy of the right ventricular free wall and septal bowing. **(D)** Short axis LGE image shows prominent enhancement at the anterior and inferior RV insertion points (asterisks).

CMR is a non-invasive, non-radiating imaging technique that allows for highly reproducible tissue characterization (90), permits assessment of radial and circumferential RV strain, and can distinguish ischemic-perfusion vs. fibrotic processes. CMR provides the best three-dimensional characterization of the RV and its dynamic relationship with the LV with high interstudy reproducibility (91). CMR also generates accurate 3D measurements of the RV throughout the cardiac cycle (92). Right ventricular mass, volume, and function can be accurately assessed and quantified on CMR. Additionally, evaluation of infiltrative disease processes relevant to development of cardiomyopathy is possible. Reduced RV ejection fraction, and RV end-systolic volume have been shown to be independent predictors of mortality (93–95). Reduced stroke volume has also been correlated with mortality (96), and improvements in stroke volume are seen in response to therapy (97, 98). Representative CMR images are demonstrated in Figure 5.

#### CMR Tissue Characterization and Perfusion Imaging

In the assessment of PH, CMR can be of particular value in patients with rheumatologic etiologies allowing for identification of occult lesions such as myocarditis, interstitial edema, myocardial infarction, and diffuse endocardial fibrosis (99). Assessment of native T1 and post-contrast T1 mapping allows for the accurate differentiation between the acute and chronic phases in many rheumatologic disorders. Understanding to what extent either ischemic injury or inflammation contributes to myocardial damage and fibrosis is also important in therapeutic interventions (100).

Late gadolinium enhancement (LGE) is a well validated approach for the evaluation of focal myocardial scarring and is the gold standard for *in vivo* assessment of replacement macroscopic fibrosis (99). CMR techniques can detect fibrosis in as little as 1 cm^3^ of tissue with excellent agreement with histologic studies (99, 101). Native T1 mapping and extracellular volume (ECV) quantification may be more sensitive than LGE techniques at detecting low-grade inflammation and diffuse myocardial fibrosis (102). In fact, in a recent study, rheumatologic patients were found to have higher T1 and T2 values, as well as expanded ECV compared with control subjects, with the most significant differences between native T1 and T2, independent of the presence of LGE (103). The extent and location of LGE in the RV can also indicate presence of RV stress. Delayed enhancement from gadolinium (10–20 mins after injection) is associated with cardiac fibrosis (104). Delayed enhancement mass at the insertion points of the RV is a sensitive and specific marker for PH (105–108). The extent of delayed enhancement mass into the interventricular septum is associated with worse RV function and clinical outcomes (109–111).

Quantification of myocardial perfusion utilizing CMR is observer-independent and highly reproducible (112). CMR perfusion imaging may allow for the investigation of characteristic disease-specific findings beyond the hemodynamic derangements in loading conditions in PH. In a study of CMR perfusion imaging in PAH patients associated with the autoimmune disorder systemic sclerosis (SSc-PAH) vs. those with idiopathic PAH (IPAH), RV and LV perfusion was significantly reduced and inversely correlated with RV workload and ejection fraction (113). Reduction in RV myocardial perfusion reserve was significantly correlated with worse hemodynamic profile and decreased RV function suggesting that reduced myocardial perfusion reserve may contribute to RV dysfunction in patients with PAH (113). CMR markers of RV remodeling and fibrosis, including RV and LV ventricular mass index, LGE and RV myocardial perfusion index, were also predictive of survival and improved with PAH-specific therapies.

#### CMR Strain Imaging

With high spatial and temporal resolution, CMR allows for quantification of global RV function across three coordinate directions (circumferential, radial, and longitudinal), as well as precise analysis of RV regional myocardial function. A variety of approaches to strain imaging with CMR are clinically available, including use of line tags and spatial modulation of magnetization (SPAMM), use of radiofrequency pulses to conduct displacement encoding with stimulated echoes (DENSE), and use of through-plane tags by strain-encoding (SENC), to name a few (114–116), although only a subset have been reliably applied to a PH population. SENC is technique with low intra- and inter-observer variabilities (117), and is based on the acquisition of two images with different frequency modulation, or low-tuning (LT) and high-tuning (HT) images in the slice-selection direction representing static and contracting tissues, respectively. Fast-SENC RV longitudinal and circumferential strain has been utilized in PH patients allowing for characterization of RV regional function with a unique pattern of reduction in RV circumferential shortening (118). Reductions in longitudinal strain correlate with RVEF and NT-proBNP in PH (119) and have a higher sensitivity and specificity to detect low RVEF when compared to circumferential strain.

Similar to STE-derived strain, CMR strain can be measured using dedicated sequences such as SENC or post-processing of cine images using feature-tracking. While CMR-derived myocardial tissue tagging and SENC have quantitative value, these modalities have not gained widespread clinical use due to expertise needed in specific sequences, additional scanning time, and the required time and cost for complex post-processing analysis (120). Ohyama et al. recently employed an alternative method of CMR strain known as multimodality tissue tracking (MTT), which similar to STE, utilizes tissue patterns obtained from cine CMR images and automatically tracks them frame to frame using an automated matching software algorithm. Findings from 30 PH patients demonstrated close correlation between MTT and SENC with high reproducibility suggesting that quantification of regional cardiac deformation using CMR cine images is feasible without the additional limitations of other CMR strain techniques. CMR and STE-basesd longitudinal strain have good inter-modality agreement while both SENC- and FT-derived circumferential strain, especially in the presence of LGE, is better detected using CMR techniques (121).

#### CMR Flow and PA Vasoreactivity

2D and 4D flow characterization through the RV is a novel technique to investigate the hemodynamics of the RV and pulmonary artery. CINE phase-contrast MRI can be used to quantify blood's velocity. When velocity in one direction is measured through a 2D plane it is called 2D flow MRI. However, it can underestimate the peak velocity if it is not orthogonal to the flow of interest and it cannot measure complex flow patterns with direction change. 4D flow MRI (3D CINE phase-contrast MRI) can analyze this through *post-hoc* 3D flow analysis (122). Flow through the pulmonary artery has been found to be qualitatively and quantitatively different in PH. Patients with PH have been found to have a reduced velocity of blood flow through the pulmonary artery correlating with higher pulmonary vascular resistance (123–126). The pulmonary artery is also noted to be less distensible in patients with PH, which may predict mortality (127–129). There is a greater retrograde blood flow through the PA in patients with PH (130) thought to be secondary to a turbulent vortex. The length of time of which the vortex is present during the cardiac cycle correlates with mPAP (131–133).

Endothelial dysfunction of the pulmonary vasculature is thought to be the central underlying pathophysiologic mechanism of PH and results in decreased relaxation of the PA (134). PA endothelial function is typically measured by invasive assessment of changes in PA in cross-sectional area and flow in response to an endothelial-dependent stress (135, 136). Previous work from our group utilizing the novel combination of 3T MRI methods with isometric handgrip exercise (IHE), a well-established endothelial-dependent stressor, demonstrated a non-invasive method of measuring coronary endothelial dysfunction with high reproducibility (137, 138). In recent work from our group, we demonstrated the feasibility of the non-invasive measurement of PA vasoreactivity in HIV patients with pulmonary vascular disease (139, 140).

## Conclusion

Echocardiography, CT, nuclear imaging, and CMR are useful for non-invasively screening, classifying, prognosticating, and monitoring effectiveness of therapy in PH. Characteristic findings for each modality are further summarized in Table 1. The standardized algorithm using echocardiogram, CT scan, and VQ scan in the initial diagnosis and classification in PH can also be supplemented by CMR methods. While multiple modalities exist and can complement each other in the investigation of PH, a well-designed clinical approach should account for expertise and availability of necessary imaging equipment and analytic software in a value-based framework focused on patient-specific clinical needs and prioritizing the minimization of imaging redundancy. Novel imaging techniques such as strain imaging, 3D echo, DECT, FDG-PET, and 4D flow MRI can evaluate for the severity of PH and can be used in conjunction with standard imaging modalities to monitor for disease progression and response to therapy. While RHC is the gold standard in the diagnosis and monitoring of PH, it can be supplemented by these non-invasive imaging modalities to ensure that it is selectively and appropriately used. Earlier detection of PA and RV dysfunction using these common imaging modalities can lead to earlier diagnosis and treatment of PH which has been shown to improve clinical outcomes.

**Table 1:** Characteristic imaging findings are summarized across imaging modalities.

**Imaging modality**	**Characteristic findings in pulmonary hypertension**
Echocardiography	**Abnormal hemodynamics**
	Right ventricular systolic pressure > 40 mmHg and/or mean pulmonary arterial pressure > 20 mmHg
	Abnormal pulmonary vascular resistance > 2 Wood Units
	Dilated inferior vena cava with or without respirophasic variation: IVC diameter ≤ 2.1 cm that collapses >50% suggests normal RAP of 3 mmHg; IVC diameter >2.1 cm that collapses <50% equivalent to RAP of 15 mmHg. In indeterminant cases, an intermediate value of 8 mmHg may be used
	Systolic flow reversal in hepatic veins suggestive of elevated right ventricular end-diastolic pressure
	**Abnormal right heart chamber size and function**
	Distortion of interventricular septal morphology suggestive of pressure volume overload
	Enlargement of the right atrium in chronically elevated right ventricular filling pressures
	Abnormal TAPSE ≤ 1.7 cm, tissue Doppler S' <9.5 cm/s, fractional area change <35%
	Presence of right ventricular hypertrophy
	Globally reduced right ventricular longitudinal strain with or without regional abnormalities
	**Abnormal regurgitant lesions**
	Presence of pulmonary and/or tricuspid regurgitation
Chest Computed Tomography Imaging	Enlargement of main pulmonary artery in comparison to ascending aorta at same level > 1
	Evaluation of lung parenchyma which may be abnormal in Group 3 pulmonary hypertension
	Assessment for acute pulmonary embolism using contrast imaging
	Assessment of chronic thromboembolic pulmonary hypertension in Group 4 disease
Scintigraphy and Nuclear Imaging	**Abnormal Ventilation-Perfusion (VQ) Scan**
	Presence of mismatched perfusion defects by VQ scan as well as signs of thromboembolism on CT and/or pulmonary angiography following three months of therapeutic anticoagulation
	**Abnormal FDG-18 uptake**
	Increased FDG-18 uptake in the right ventricle and pulmonary artery
Cardiac Magnetic Resonance Imaging	**Abnormal right heart chamber size and function**
	Increased right atrial and ventricular volumes
	Abnormal interventricular septal morphology suggestive of pressure/volume overload
	Presence of right ventricular hypertrophy
	Reflux of contrast into the hepatic veins
	Decreased right ventricular function
	Abnormal CMR-derived strain along both longitudinal and circumferential axis
	**Abnormal tissue characterization**
	Abnormal native T1 mapping and expanded extracellular volume suggestive of tissue inflammation seen in acute phase
	Presence of late Gadolinium enhancement which can be seen at insertion points of the right ventricle and within the right and left ventricles
	Suggestive of fibrosis and tissue remodeling
	**Abnormal perfusion**
	Reduced right and left ventricular perfusion is inversely correlated with pulmonary pressures, and right ventricular workload and ejection fraction
	**Abnormal flow and pulmonary arterial vasoreactivity**
	Reduced pulmonary arterial blood flow velocity correlates with increased pulmonary vascular resistance
	Decreased pulmonary arterial distensibility
	Abnormal pulmonary artery vasoreactivity suggestive of endothelial dysfunction

## Author Contributions

MM was the principal investigator, had access to all the data in the study, and takes full responsibility for the integrity and accuracy of the manuscript. All authors contributed equally to the design, drafting, and final approval of this manuscript.

## Funding

Funding for this work was supported by the American Lung Association (AB), NIH/NHLBI R01HL147660-03 (AH), NIH/NHLBI R01-HL114910 (PH), NIH/NHLBI K23-HL146889 (SH), Jerome Green Foundation (SH), Scleroderma Foundation (MM and SM), Department of Defense W81XWH2010768 (SM), and Johns Hopkins Clinician Scientist Award (MM).

## Conflict of Interest

The authors declare that the research was conducted in the absence of any commercial or financial relationships that could be construed as a potential conflict of interest.

## Publisher's Note

All claims expressed in this article are solely those of the authors and do not necessarily represent those of their affiliated organizations, or those of the publisher, the editors and the reviewers. Any product that may be evaluated in this article, or claim that may be made by its manufacturer, is not guaranteed or endorsed by the publisher.
